# Are (All) Consumers Averse to Bitter Taste?

**DOI:** 10.3390/nu11020323

**Published:** 2019-02-02

**Authors:** Riccardo Vecchio, Carla Cavallo, Gianni Cicia, Teresa Del Giudice

**Affiliations:** Department of Agricultural Sciences, Università degli Studi di Napoli Federico II, 80055 Portici, Italy; riccardo.vecchio@unina.it (R.V.); cicia@unina.it (G.C.); agriqual@unina.it (T.D.G.)

**Keywords:** hedonic liking, experimental auctions, willingness to pay, broccoli pesto

## Abstract

The current study combined hedonic liking with non-hypothetical experimental auctions to measure consumer preferences for bitter tasting food and identify individual socio-demographic and psychographic characteristics that influence bitter aversion. Furthermore, the research analyzed whether consumer preferences for bitter food were influenced by sensory and health-related information. Findings reveal that respondents (*N* = 205) are not averse to bitter taste; while, socio-demographic traits influence bitter acceptance, as higher education level and gender (female) positively affect preferences, together with specific individual characteristics as high compensatory health beliefs. Moreover, results prove that participants positively respond to health-related information, whereas information on bitterness-taste generates lower preferences.

## 1. Introduction

Antioxidants represent a fundamental category of healthy substances that are able to prevent many metabolic diseases [1,2]. These compounds, very often, give the food a bitter taste [3]. This is the case of vegetables from the Brassicaceae family containing glucosinolate hydrolysis products, sulforaphane and indole-3-carbinol [4]. Their effect on the prevention of several types of diseases seems to be substantial and more powerful than general fruit and vegetable consumption [4,5].

Plants produce toxic substances that tend to be bitter as a defense strategy from herbivores [6]. This is the reason why during evolution humans learnt to avoid this sensory property in food as it was a danger signal [3,7]. There are several bitter substances that deviate from this bitter-poison link, such as peptides in meat and cheese with multifold effects on human nutrition. The interest of this article is devoted to peculiar bitter substances with healthy properties that are present in Cruciferae (glucosinates) [8]. Bitterness also characterizes other healthy substances in other foods, as: EVOO [9]; saffron [10]; whole wheat bread [11] and tea [12].

In particular, the bitter taste of antioxidants compounds is addressed as being the main cause of rejection of *Cruciferae* by consumers [13,14]. This is leading the food industry and breeders to lower the content of bitter, and thus healthy, substances from these products [3,15]. At the same time, since exposure is one of the main powerful elements in lowering the avoidance toward bitter taste [16], bitter taste is also constantly dropping its popularity among consumers [17,18].

Nevertheless, there can be some exceptions to this general tendency which can be represented by individual differences among consumers. In fact, the level of bitterness detected by humans in food has a source of variability that is genetically determined [19]. This trait is believed also to be an indicator of overall taste sensitivity and it is supposed to influence consumers’ perceptions and preferences and, in turn, to influence their diet [19,20].

Other deviations from bitterness rejection can occur when consumers attach a positive meaning to the bitterness feature. For example, in products such as chocolate, coffee or alcoholic beverages, due to the goals that are connected to their consumption, the consumer overcomes innate rejection and appreciates the presence of the bitter taste [21,22,23,24], reaching the point that a preference for bitterness becomes mainstream for such products [25,26].

In this context, the aim of the present research is to investigate consumer preferences for bitter tasting *Cruciferae* and identify individual socio-demographic and psychographic characteristics influencing bitter avoidance/acceptance. Furthermore, the study analyzes whether consumer preferences for bitter food are influenced by sensory and health-related information. Broccoli pesto was selected as a case study considering that broccoli is among the most consumed vegetables in the geographical area of analysis (Southern Italy) and that pesto is very popular among individuals of different social classes.

## 2. Materials and Methods

Adapting the approach proposed by Combris and colleagues [27], the current study combines features of hedonic tests with those of economic experiments to measure consumer preferences for different food characteristics and the way in which sensory and health information influence these preferences. Non-hypothetical experimental auctions were used to elicit individual preferences (recording monetary values) in order to avoid problems related to hypothetical bias [28], while hedonic ratings were applied to measure the respondent’s blind and expected liking. Experimental auctions use real products and real money and are designed to induce each participant to submit a bid that sincerely reflects her/his value for one unit of the auctioned goods [29]. Therefore, experimental auctions mimic a real market scenario, allowing researchers to determine the monetary value people place on specific goods. However, from the perspective of a research participant, the experimental auction procedure presents individuals with the unfamiliar problem of being asked to provide the highest price that they would be willing to pay for one or more (often novel) goods [30]. Consequently, experimental auctions are anticipated by training rounds in which the participants are shown, in a very easy way, the simple task that they are required to perform.

### 2.1. Stimuli

In order to understand the product to be used as a case study for the current analysis, four focus groups (*N* = 38) were conducted with consumers of different age categories (ranging from 18 to 64 years old). The focus groups were aimed at investigating the opinions of consumers toward bitter taste in general and toward particular products characterized by this flavor. The discussions were also extended to participants’ habits in terms of cooking and eating to uncover potential, privileged targets for the subsequent study. The focus group data analysis suggested that the product that was best fitting with the research objectives was broccoli. Indeed, previous literature has identified vegetables belonging to the family of *Cruciferae* as a model food for bitterness since, according to consumers, broccoli is, on average, more bitter than other vegetables [31]. Furthermore, agreeing with previous studies, broccoli is the most consumed and appreciated product within the *Cruciferae* family in Southern Italy [32] where there are also locally grown typical bitter *Cruciferae* cultivars [33]. In addition, since the exposure of consumers from Southern Italy to this bitter vegetable is medium to high, consumers’ avoidance toward this product is expected to be lower compared to other foods [3]. This made Southern Italy a suitable case study, also considering that this place has been the establishing point of the Mediterranean Diet, which contains a large share of vegetables over other food groups [34,35,36].

Finally, this product is widely recognized by consumers as very healthy, being an important prevention factor against several diseases and other conditions [37]. Previous literature suggested that when healthy goals are very salient in the mind of the consumers, they can be willing to compromise taste for healthiness, leading to another motivation for analyzing preferences for bitterness via a broccoli-based food product [38,39].

Specifically, three experimental products, i.e.; broccoli pesto, were produced for the specific aims of the research by a private food company. The choice of pesto was motivated by the possibility to include this food in many dishes without particular cooking knowledge, and because pesto is popular among individuals of different social classes [40,41]. The three broccoli pesto were produced by one factory and on the same day, according to the same recipe, only differing in the percentage of broccoli leaves that were contained in the final product, being that leaves are more bitter compared to sprouts (Table 1). This difference leads to diverse bitterness of the goods. Indeed, the three broccoli pesto were evaluated by a professional sensory panel assigning a level of bitterness to each on a scale from 1 (extremely low) to 9 (extremely high), respectively of 1 (hereafter called LOW), 4 (hereafter called MEDIUM) and 7 (hereafter called HIGH).

### 2.2. Experimental Procedure

The experimental auctions protocol was developed following well-established guidelines [29,42]. In particular, the random *n*th price auction [43] with the full-bidding approach was applied. In this procedure, the core features of the Becker–DeGroot–Marschak (BDM) mechanismmechanism and second price auction are combined, obtaining an endogenous price of the market (as in a second price auction), however it is determined in a random way (like the in the BDM mechanism). Specifically, all bidders simultaneously submit sealed bids for all the auctioned goods, then each bid is rank-ordered from highest to lowest; a random number is subsequently drawn between 2 and the number of participants in the experimental session (the *n*), then finally, one unit of the good is sold to each of the (*n*−1) highest bidders at the *n*th-price [43]. As noted by Shogren and colleagues [43] randomness is used to effectively engage all bidders, providing all participants a positive probability of being a purchaser of the auctioned good. This type of auction was selected as it allows all bids to influence the results of the auction and thus also effectively engages participants with lower interest towards the goods as even low bids could become binding and thereby lead to a large percentage of participants purchasing the auctioned products. This mechanism is incentive compatible, meaning that subjects have an incentive to truthfully reveal their value for all the auctioned products [44]. Among the core advantages of experimental auctions over other incentive-compatible value elicitation methods, we should highlight that the auctions deliver willingness-to-pay (WTP) for each individual participant, avoiding the need to make parametric assumptions about the shape of the market demand curve [45], and also grants participants maximum freedom compared to other mechanisms in which participants can only accept/reject the price of a product described as a bundle of attributes [46].

**Figure 1:**
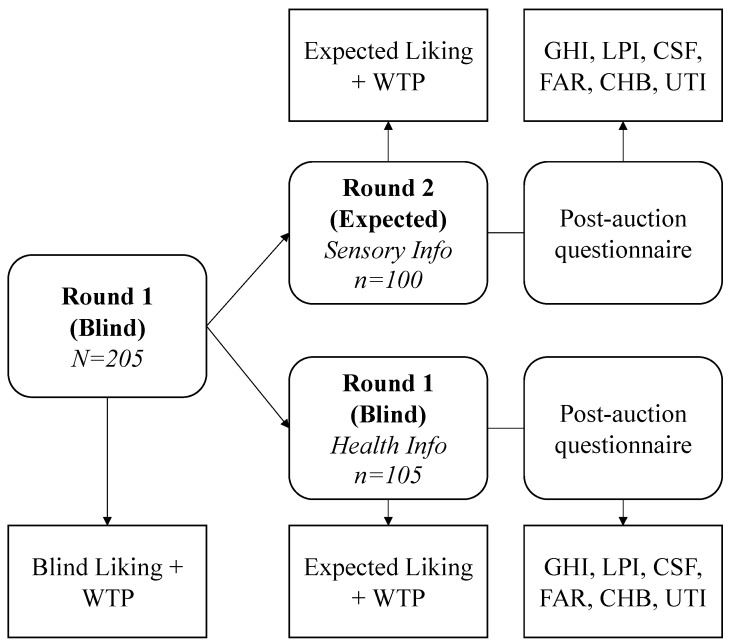
Experimental design. WTP (willingness-to-pay), GHI (General Health Interest), LPI (Light Product Interest), CSF (Craving for Sweet Foods), FAR (Food as A Reward), CHB (Compensatory Health Beliefs), UTI (Unhealthy=Tasty Intuition).

In the current study, a hybrid within-subject and between-subject design was implemented, i.e. all participants (*N* = 205) performed the same tasks and received the same information in round 1 (blind round), whereas in Round 2 (expected round), half the sample was provided with an information treatment on the bitterness level of the products (i.e. sensory) and half the sample received information on the health properties of the goods (Figure 1). A total of 18 experimental sessions were performed on six consecutive weekdays and throughout the morning and afternoon hours, with 10 ± 2 participants each, lasting approximately 65 minutes. Individuals were compensated for their time with 20€ cash. The entire experimental flow involved seven consecutive steps: (1) participants were welcomed into the experimental laboratory, were asked not to communicate with each other and to complete the informed consent and received the participation fee; (2) the procedure of computerized sealed-bid random *n*th experimental auction was fully explained and training rounds with chocolate bars were performed; (3) participants blind tasted the three products one by one and expressed their overall liking on a 9-point hedonic scale [47] and WTP for a 100 gram jar; (4) participants were randomly assigned to one of the two experimental conditions: healthiness or bitterness information treatments and expressed their expected liking (9-point hedonic scale) and WTP for the same three products; (5) post-auction survey (drawn on previous food consumer research and economic theory); (6) one round of auctions, one product and one price were randomly drawn by a participant, the products were then sold to the winners (to avoid demand reduction effects). The post-auction questionnaire and the tasting procedure were pretested with a small group of consumers (*n* = 12) to avoid ambiguity and effects from the information treatments’ wording. All procedures that were performed in the studies involving human participants were in accordance with the ethical standards of the institutional and/or national research committee and with the 1964 Helsinki declaration and its later amendments or comparable ethical standards. Informed consent was obtained from all individual participants that were included in the study.

### 2.3. The Survey

From an analysis of literature, we developed a set of socio-demographic characteristics of consumers that was supposed to be able to influence perception and liking of bitter products. According to that, the post-auction survey was structured.

We started from general questions about purchase and consumption habits of consumers in order to understand their familiarity and involvement with vegetables as a product [48,49]. Then, we asked respondents about their current level of hunger, as some motivational states are believed to affect food preferences [50].

Since, in the case of the investigated products, we have a gap between taste and healthiness, we used scales that were developed to understand to what extent the respondents are willing to trade taste for health benefits in the food that they usually eat. The seven scales are: General Health Interest (GHI), Light Product Interest (LPI), Natural Product Interest (NPI). Craving for sweet foods (CSF), Food as a reward (FAR), Compensatory Health Beliefs (CHB), Unhealthy=Tasty Intuition (UTI). Specifically, the Health and Taste Attitude Scales (composed of three sub-scales [51]) are directed at measuring the importance that is attached to sensory and health aspects in food choice. On the other hand, the Unhealthy=Tasty Intuition [39] is able to understand to what extent these two aspects are considered antithetical. Besides, Compensatory Health Belief (CHB, [52]) is the scale that measures how the respondent is indulgent in the case of unhealthy behaviors.

Nevertheless, we considered a physiological difference among humans: individuals have different levels of taste sensitivity that are genetically determined, and this is especially true for bitter taste [19]. This difference leads consumers to eat more or less bitter foods, so it is able to shape either the individuals’ diets and their bitterness exposure. Exposure, in turn, can shape bitterness liking. So, understanding the taste sensitivity can help to disclose important antecedents to bitterness acceptance. To this purpose, we measured taste sensitivity of participants through the tasting of a solution made with 6-n-propyl-thiouracil (PROP) at the end of the experiment. This solution is plain, slightly bitter or extremely bitter according to the personal level of taste sensitivity, so we asked participants to rate the bitterness of this solution on a scale from 1 to 9. At this point, it was possible to categorize consumers in the subsequent groups: non-tasters (rating from 1 to 3), medium tasters (rating from 4 to 6) and super-tasters (rating from 7 to 9). At the end, general demographics (age, gender, living area, etc.) closed the questionnaire.

### 2.4. Information Treatments

**Figure 2:**
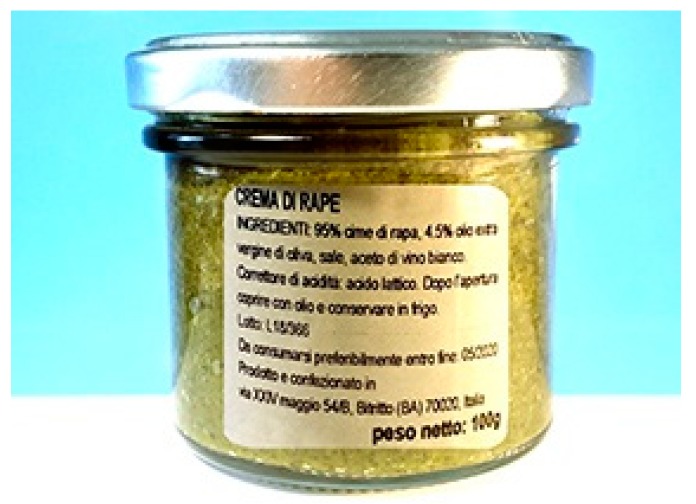
Auctioned Broccoli pesto jar. Note: Beyond the ingredient list (top), the label reported the best before consumption date, producer’s address and net weight (bottom).

In the blind tasting round (Round 1), the products were offered to individuals one at a time on a white plastic spoon containing 4 grams of product and, for each participant, a package of unsalted crackers was placed on a small paper plate together with a bottle of still spring water. The order of samples was randomized across sessions according to a Latin square that was balanced for order and first-order carry-over effects [53]. Participants were also asked to rinse their palate with still water and eat a cracker for neutralization between tasting different samples. To increase realism, pesto jars were handed out to participants before collecting the bids. To prevent the influence of brand, such as packaging and other external cues, the three jars were provided with a label containing only the legally required information (Figure 2).

In the second round (expected liking), two types of information were distributed via personal monitors and read aloud by one researcher. Specifically, 100 participants received sensory information on the level of bitterness of each jar of pesto: low, medium and high (sensory information treatment), while 105 participants received information on the amount of polyphenols content of each good (low, medium and high) and the relation between polyphenols and personal health (health information treatment). Randomization of participants to the information treatments was successful as all socio-demographic characteristics are balanced over the subgroups, according to statistical analysis.

### 2.5. Sample Characteristics

A central location test was performed in Naples (Southern Italy) where 205 participants were recruited by a non-profit association according to two eligibility criteria: consumers of vegetables and at least partially responsible for the family’s grocery shopping. In addition, participants were asked not to smoke, eat or drink anything, except water, at least 1 hour before their participation in the experiment to avoid any influence of their taste perception from prior smoke/food/beverage consumption. Participants signed an informed consent prior to their participation. In terms of demographic data, 76% of respondents lived in an urban area, the mean age was 34.2 years old (SD 13.4), 63% were females, the average number of household members was 3.6 and their income was in line with the national average (the lower interval was less than 2.400€, the middle interval was between 2.400€ and 3.400€ and the upper interval was more than 3.400€ intervals were selected considering the national average provided by ISTAT [54]). Most of respondents are within a normal weight, are not on a diet and are not hungry at the moment of the experiment (Table 2). Moreover, the majority of participants have a high taste sensitivity which was measured through the PROP test.

In addition, individual psychographic traits were recorded to further investigate the consumers’ heterogeneity (reported in Table 3). On average, participants declare to be medium-to-highly interested in personal health (4.85 mean score out of 7 for General Health Interest), they are moderately appealed by light products (3.8 out of 7 for Light Products Interest), reasonably interested in natural products (4.42 out of 7 for Natural Products Interest), highly appealed to sweet foods (5.4 out of 7 for Craving for sweet foods) and have a medium-to-high tendency to use food as a reward (4.47 out of 7 for Food as a reward). Furthermore, the sample is, on average, not convinced by trade-offs between healthy and unhealthy habits (2.57 out of 7 for Compensatory Health Beliefs) and by the intuition that what is tasty is always unhealthy (2.85 out of 7 for Unhealthy=Tasty Intuition).

### 2.6. Data Analysis

To formally test whether the WTP for the three products were different during the first blind round, we conducted a series of complementary statistical tests on the equality of means, samples distribution and the equality of distributions. For the auction data, we followed the common practice that was used in similar studies and estimated a Tobit model censored at zero [29]. To increase the power of the statistical tests with the econometric model, we pooled the observations on WTP across the three products. Hence, the sample size for this fitted model was 615 (205 × 3). Specifically, to identify the factors that influenced WTP, we estimated a Tobit model where WTP*_ji_* is the latent value of WTP for broccoli pesto type *j* and subject *i*, expressed as a function of pesto type and individual characteristics, and the individual specific disturbance term for subject *i* is u*_i_*. The final model is:

WTP*_ji_* = *β*_0_ + *β*_2_ Pesto*_j_* + *β*_3_ age + *β*_4_ female + *β*_5_ BMI + *β*_6_ education + *β*_7_ financial situation + *β*_8_ market purchaser + *β*_9_ hunger + *β*_10_ super-taster + *β*_11_ hedonic liking score + *β*_12_ LPI + *β*_13_ CSF + *β*_14_ FAR + *β*_15_ CHB + *β*_16_ UTI + *β*_17_ GHI + *β*_18_ NPI + u*_i_*.

All data analyses were carried out using STATA 15.

## 3. Results

### 3.1. Bitter Preferences and Drivers in Blind Liking Round (Round 1)

**Figure 3:**
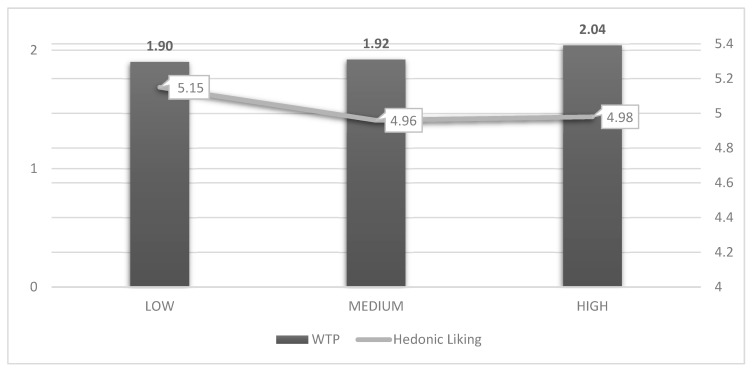
Blind round, mean WTP (€) and hedonic liking scores.

Mean hedonic scores and WTP that were expressed for the three products in the blind round suggest that consumers equally liked the products as no statistically significant differences among the three pesto are detected (Figure 3).

Data from the first round were further analyzed through a Tobit model in order to understand which consumer characteristics are able to characterize the different WTP. Table 4 reports the estimated coefficients (and standard errors in brackets). The analysis was performed stepwise, thus the effect of each variable was tested and, in the end, only statistically significant relations were kept in the final explanatory model. Hence, a smaller set of variables is present in the model compared to the ones that were collected with the experiment and the questionnaire.

On average, WTP for the HIGH product were higher compared to the other two pesto. A higher WTP was registered for consumers with specific socio-demographic characteristics: individuals with a higher education level and participants who were hungry at the moment of the experiment. Whereas, male respondents expressed lower WTP compared to females. Furthermore, higher WTP were made by super-taster respondents, by individuals that scored high on the Light Product Interest (LPI) scale, on the Food as a reward scale (FAR) and on the Compensatory Health Belief scale (CHB). Not surprisingly, hedonics liking scores also had a positive effect on WTP. Conversely, WTP were negatively influenced by higher scores on the Craving for sweet foods (CSF) scale and by the unhealthy=tasty intuition (UTI) index. Finally, respondents that regularly buy vegetables in local markets expressed lower WTP for the auctioned products.

### 3.2. Information Treatment’s Effects on Consumer Preferences (Round 2)

**Figure 4:**
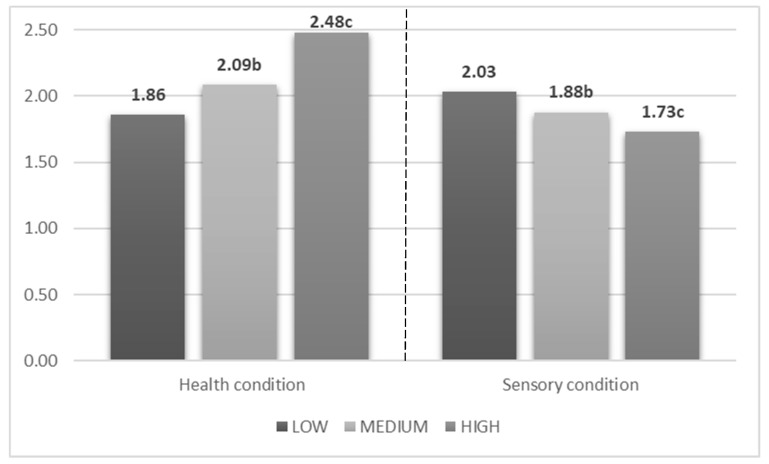
Mean WTP (€) in the Expected round. Note: Mean bids with different superscripts are significantly different according to a Wilcoxon test for paired samples (*p* < 0.05) within the information treatments.

Considering data from the second round’s (Expected liking) findings revealed different mean WTP among the two information treatments (Figure 4). In the health information treatments, products communicated as having higher polyphenols (MEDIUM and HIGH) content received statistically higher WTP. While, in the sensory treatment (i.e. information on bitter taste scores are conveyed), the opposite occurs, revealing participants’ positive response to health-related information and negative expectations from a bitter-tasting food.

## 4. Discussion

Consumers’ preferences are affected by many factors; even when sensory evaluations reveal high liking scores, extrinsic characteristics can strongly impact final choices. The present study combined hedonic liking with non-hypothetical experimental auctions to measure consumer preferences for bitter tasting food and identify individual socio-demographic and psychographic characteristics related to bitter avoidance/acceptance, also analyzing whether consumer preferences for bitter food are influenced by sensory and health-related information.

Mean WTP reveals that respondents are not averse to bitter taste, as no statistically significant difference was detected in the blind liking scores and in WTP among the three broccoli pesto (Round 1). This result was somewhat unexpected as there is ample literature that demonstrates that, among consumers, there is a wide aversion toward bitter taste [17,55,56,57]. The previously underlined exceptions were defined for particular foods, such as chocolate or coffee [23,58,59], especially in the presence of specific motivational states [50]. Nevertheless, the explanatory model revealed an additional detail: not only that bitterness was not disliked, however that among the tested products, the most bitter received the higher bids. This confirms that our research disclosed a further exception to the widespread avoidance from consumers toward bitterness in food.

The reasons behind this bitterness acceptance are still unclear. Nevertheless, there is no evidence that bitterness acceptance can be related to the physiology of the sample of respondents. In fact, the wide majority of consumers were identified as supertasters (Table 2); furthermore, the explanatory model showed that, on average, supertasters’ bids were higher than the ones that were expressed by the other groups of participants (Table 4). Besides, the sample contained a wide share of young consumers who generally tend to be averse to bitter taste [60]. There is room to suppose that reasons rely on socio-cultural factors. In our study, some socio-demographic traits are related to bitterness acceptance, for example, higher education level and gender (female) characterize respondents who showed preference for bitter products, together with specific individual characteristics, such as high compensatory health beliefs.

The element that led consumers to increase their bids for more bitter products may be the healthiness of the investigated products. In fact, characteristics of consumers that were collected with the questionnaire showed that the tested sample was made, on average, by respondents who were tendentially concerned for their health, fond of natural food and who were convinced that healthy food tastes better (Table 3).

The hypothesis that healthiness plays a pivotal role in judgements of consumers can be further confirmed by the second part of the research. In fact, results from round 2 of the experiment (Expected liking) proved that participants positively responded to health-related information (increasing WTP and liking score), while information on bitterness generates lower preferences. This result is in line with previous research; in fact, literature suggests that consumers use the word bitter with a negative meaning, preferring synonyms in positive contexts [61], therefore, the tested sensory message generated negative expectations in consumers. Meanwhile, the effectiveness of health-related messages in generating positive expectations has already been shown in previous studies [62,63,64]. Especially when healthy goals are very salient in the mind of consumers, they can be willing to compromise taste for healthiness [38,39]. This finding has been reinforced in our study by the estimation of a model which yielded that consumers, after a healthy framing, did raise their WTP in a significant way only for the most bitter product (HIGH).

A possible explanation for consumers lowering their WTP within the taste message framing can be found in selective attention [65], as consumers do not like bitterness and use the word bitter with a negative meaning, so the message about bitterness can lead them to concentrate on such a feature and lower their ratings. Moreover, current results demonstrate the importance of attaching positive information to products with a low/poor image (i.e. broccoli), as information affects taste beliefs and, in turn, has a large impact on selection behavior [66]. This result could yield practicable suggestions for promotion strategies that are aimed at increasing the popularity for bitter tasting products. In fact, we believe that salient information about health can also contribute to generate positive expectations in healthy products that are not preferred by the majority of the population. Due to the effect of exposure, consumers can get accustomed to the taste and learn to appreciate it, just as what happened in the case of bitter chocolate [59].

Limitations of the current study lie in the narrow area of research with homogenous food traditions and the focus on one specific product (broccoli pesto), while a national or cross-national study performed on multiple foods would provide a broader view of preferences of consumers toward bitter taste, not being strictly linked to the features of the case study product. Then, a research conducted on a sample that better represents the age distribution of the population would yield more detailed results about the reaction of consumers belonging to different age groups. Furthermore, the present study investigated only two types of information treatments (health and sensory info), whereas different messages as well as non-traditional framings, such as nudging-based techniques, could be employed and tested.

## Figures and Tables

**Table 1:** Experimental products’ features.

Product	Coding	Ingredients	Bitterness Level
100 gram jar of broccoli pesto #1	LOW	95% broccoli (75% sprouts, 25% leaves), 4.5% extra virgin olive oil, salt, white wine vinegar and lactic acid.	1
100 gram jar of broccoli pesto #2	MEDIUM	95% broccoli (50% sprouts, 50% leaves), 4.5% extra virgin olive oil, salt, white wine vinegar and lactic acid.	4
100 gram jar of broccoli pesto #3	HIGH	95% broccoli (25% sprouts, 75% leaves), 4.5% extra virgin olive oil, salt, white wine vinegar and lactic acid.	7

**Table 2:** Characteristics of participants.

Variable	*n*
**Age**	
<25	60
25–34	81
35–44	17
45–54	19
55–64	25
>64	3
**Gender**	
Female	130
Male	75
**Education**	
High school diploma or lower	78
Degree	105
Post-degree	22
**Body Mass Index (BMI)**	
Underweight	5
Normal weight	141
Overweight	42
Obese	17
**Currently on a diet**	
No	172
Yes	33
**Financial situation**	
Worse than the national average	16
In line with the national average	163
Better than the national average	26
**Preferred groceries sale channel**	
Hyper/supermarket	51
Small shop	79
Direct sale	23
Local Market	31
Farmers’ market	21
**Level of hunger at the time of the experiment**	
Low	109
Medium	66
High	30
**Taste Sensitivity (PROP test)**	
Non-tasters	21
Medium tasters	25
Super-tasters	159

**Table 3:** Participants’ psychographic characteristics.

	Mean	St. Dev.
General Health Interest (GHI)	4.85	1.06
Light Product Interest (LPI)	3.80	1.17
Natural Product Interest (NPI)	4.42	1.03
Craving for sweet foods (CSF)	5.40	1.20
Food as a reward (FAR)	4.47	1.39
Compensatory Health Beliefs (CHB)	2.57	0.71
Unhealthy=Tasty Intuition (UTI)	2.85	1.54

Note: All scales range from 1 (low) to 7 (high).

**Table 4:** Selected parameter’s estimates of the Tobit model for WTP collected during the blind round (Round 1).

Variable	Coefficient
**Auctioned products (reference category: Low)**	
MEDIUM	0.09 (0.11)
HIGH	0.21 * (0.10)
**Socio-demographics**	
Male	−0.26 *** (0.09)
Education	0.11 ** (0.04)
**Individual characteristics**	
Local market purchaser	−0.24 * (0.13)
Level of hunger	0.09* (0.04)
Super-taster	0.05 ** (0.02)
Hedonic Liking Score	0.29 *** (0.03)
Light Product Interest (LPI)	0.13 *** (0.04)
Craving for sweet food (CSF)	−0.11 *** (0.04)
Food as a reward (FAR)	0.06 * (0.03)
Compensatory Health Beliefs (CHB)	0.21 *** (0.05)
Unhealthy=Tasty Intuition (UTI)	−0.07 ** (0.03)
**Constant**	−0.99 ** (0.48)

Note: Asterisks represent statistically significant coefficients at the levels: * *p* ≤ 0.1; ** *p* ≤ 0.05; *** *p* ≤ 0.01.
